# Notes and Notices

**Published:** 1887-03

**Authors:** 


### NOTES AND NOTICES.

PROFESSOR KENT'S LECTURES.—In the February number we published a lecture by Professor Kent upon Eupatorium Perfoliatum. By an editorial blunder we credited the report to Dr. C. S. Durand, when it was really prepared by Dr. Frank Kraft, now on the editorial staff of *The Medical Advance*

THE MEDICAL ADVANCE.—January number, criticising our heading of Dr. Kent's lectures, says: "Stenographically reported lectures are *usually* extemporaneous, since none but the veriest tryo in short-hand would attempt to follow a rapidly spoken lecture when the professor held the manuscript, which could be had for the asking. [This one is on you, Mr. HOMŒOPATHIC PHYSICIAN.]"

All right, Mr. MEDICAL ADVANCE! We are "laying for" you! We won't tolerate criticism. And though we have expunged *extemporaneous* from our heading it is not because of *your* criticism; oh! dear, no! By the way, we don't find *tryo* in the dictionary. The word there appears to be TYRO. And here's one on you, Professor Allen.

INDIANA INSTITUTE OF HOMŒOPATHY.—The twenty-first session of this Society will be held at Indianapolis in May. The principal officers for 1887 are: President, J. A. Compton, M. D., Indianapolis; Vice-President, Z. Hockett, M. D., Anderson; Second Vice-President, J. R. Haynes, M. D., Indianapolis; Treasurer, J. S. Martin, M. D., Muncie; Secretary, William B. Clarke, M. D., Indianapolis.

INTERNATIONAL HAHNEMANNIAN ASSOCIATION.— The seventh annual meeting of the International Hahnemannian Association will be held at Long Branch, New Jersey, next June. The date will be given in a subsequent notice.

INTERNATIONAL HAHNEMANNIAN ASSOCIATION.—The Executive Committee of the International Hahnemannian Association at their next meeting should provide room for "reports from delegates," that all Hahnemannian Societies throughout the country should be encouraged to send representatives. We are informed that the local Hahnemannian Society of Rochester has set the example by electing a delegate to the next meeting.

